# Implementation of multi-mode nursing insulation program for patients receiving surgery for spine tumor: a propensity score-matched analysis

**DOI:** 10.1186/s12893-021-01463-1

**Published:** 2022-01-08

**Authors:** Juan Liu, Chunyan Gao, Hailong Fu, Xiaonan Zhou, Li Zhang, Xiaomei Tang, Yanru Wu, Hui Zhu, Sisi Yang, Yafeng Qu, Yajuan Yang, Haiqin Yang

**Affiliations:** Changzheng Hospital, Affiliated to Naval Military Medical University, Shanghai, China

**Keywords:** Spine tumor, Enhanced recovery after surgery, Hypothermia, Multi-mode nursing insulation program, Propensity score-matched

## Abstract

**Background:**

Spinal tumor surgery usually involved long operation time, large area of soft tissue resection and long wound, and was prone to hypothermia during the operation. Therefore, actively promoting insulation and optimizing the intraoperative insulation program have great potential in reducing the incidence of hypothermia and reducing the incidence of postoperative complications. In this study, we compared patients who did not implement multi-mode nursing insulation program (MNIP) with those who implemented MNIP, observing and comparing clinical outcomes, and complications in both groups, with the aim of developing an optimal management plan for the preoperative, intraoperative, and postoperative periods, respectively.

**Methods:**

We selected 2 periods of 1 year, before (n = 120 patients) and after MINP implementation (n = 120 patients). Data were collected on patient demographics, operative, perioperative details, temperature changes, anesthesia recovery effect, incidence of postoperative wound infection, length of hospital stay and complications. PS analyses were used for dealing with confounding bias in this retrospective observational study.

**Results:**

After PS matching, the outcomes of 120 well-balanced pairs of patients were compared (No-MNIP vs MNIP). There was no significant difference concerning the satisfaction survey. The results indicated that the MNIP had better insulation effect at 90 min, 120 min, 150 min after anesthesia induction and after surgery. There were 16 cases of complications in the No-MNIP group and 5 cases in the MNIP group postoperative, which have significant statistical difference.

**Conclusion:**

In this study, the incidence of intraoperative hypothermia was effectively reduced by adopting the multi-mode insulation scheme, thus reducing the incidence of incision infection and shortening the length of hospital stay of patients.

## Background

With the continuous improvement of the average life expectancy of the population, the incidence of spinal tumors is increasing year by year. Spinal tumors are mainly divided into primary tumors and metastatic tumors. Primary spinal tumors are rare, accounting for about 4.6 to 8.8% of all bone tumors, and spinal metastases account for 50% of all bone metastases. It has been reported in the literature that 10 to 30% of patients with primary malignant tumors will have spinal metastasis at an advanced stage. Intractable pain, spinal instability and nerve compression are the most common symptoms of spinal tumor. 30% of patients with spinal tumor will seriously affect the quality of life, have paraplegia and even shorten the survival period. The main goal of surgical treatment is to remove the tumor, relieve nerve compression and rebuild the stability of the spine. Spinal tumor surgery usually involved long operation time, large area of soft tissue resection and long wound, and was prone to hypothermia during the operation [1–6].

Perioperative hypothermia means that the core body temperature of surgical patients was lower than 36 ℃, and the incidence was about 50–90%. It could increase the infection rate of surgical incisions, the incidence of postoperative complications, and prolong the patient’s hospital stay. In United States, postoperative infection had become the 4th leading killer, and its death toll exceeded the combined number of AIDS, breast cancer and traffic accidents. Its annual medical expenses were 1–10 billion dollars. CMS-SCIP proposed the best strategy to prevent surgical site infections: proper hair removal, reasonable preventive use of antibiotics, maintenance of normal body temperature, and blood glucose control. It could be seen that maintaining a normal body temperature during the perioperative period had an important significance in preventing infections at the surgical site. *Huang* conducted a survey of 28 research centers and found that passive thermal insulation measures were less than 90% and active thermal insulation measures were less than 10% in various stages of thermal insulation methods. The incidence of hypothermia during the perioperative period of general anesthesia patients was as high as 44.5%. Therefore, actively promoting active insulation and optimizing the intraoperative insulation program have great potential in reducing the incidence of hypothermia and reducing the incidence of postoperative complications [7–14].

Enhanced Recovery After Surgery (ERAS) is the core ideology leading the development of field of surgrical treatment [15–19]. However, there is still no idea about the application of the ERAS concept in nursing insulation program. Our hospital began the implementation of a multi-mode nursing insulation program (MNIP) regarding the development of ERAS for patients receiving surgery for spine tumors in September 2019. The MNIP aimed to minimize and avoided the occurrence of hypothermia during spinal tumor surgery, and minimized infection and orther complications.

In this study, we used propensity score-matched (PS) analysis to compare the controlled group patients MNIP with those patients received MNIP, observing and comparing clinical outcomes, and complications, and also aimed to develope an optimal treatment plan.

## Patients and methods

### Inclusion and exclusion criteria

The study was approved by the Institutional Ethics Committee of Changzheng Hospital. The inclusion criterias were as follows: (1) patients were diagnosed as with spinal primary malignant tumors or metastatic tumors; (2) survival is expected to be longer than six months. The exclusion criterias were as follows: (1) patients with malignant metastatic tumors who cannot tolerate surgery or undergo palliative surgery; (2) having metastases in the lungs or other organs; (3) survival is expected to be less than six months. All the surgeries were performed by the same group of surgeons.

The MNIP multi-disciplinary team comprised of spine surgeons, anesthesiologists, physiotherapists, and nurses finalized the MNIP. This led us to selected 2 periods of 1 year, before (Group No-MNIP, from May 2019 to Apr 2020, n = 120 patients) and after implementing of the ERAS (Group MNIP, from May 2020 to Apr 2020, n = 120 patients).

### Multi-mode nursing insulation program

Before the patient arrived in the operating room, the room temperature was preheated at 22–24 °C, and the specially-made segmented nine-holes with a length of 1.2 m and width of 1.0 m were preheated and covered to keep warm; the pre-heated segmented thermal insulation surgical gown was used during the operation. Used air heaters to heat and inflatable heat preservation, adjust at any time according to the patient's body temperature, to ensured that the patient's body temperature was above 36 °C; used an infusion warming instrument for infusion; recorded the disinfection time, the amount of input and output, the temperature of the rinsing solution, and implement targeted preventive measures for patients who were prone to hypothermia. Active warmth and passive warmth were synchronized; body thermometers were continuously monitored.

### Evaluation outcome

Patient demographics, clinical history, comorbidities, and operative details were noted from the hospital records. The outcome measures for the study were as following:

#### Temperature changes during surgery

The rectal temperature was recorded after induction of anesthesia (T1), 30 min (T2), 60 min (T3), 90 min (T4), 120 min (T5), 150 min (T6), and 1 day after operation (T7).

#### Evaluation of anesthesia recovery effect

The Steward recovery score was used to evaluate the anesthesia recovery effect of the two groups of patients after the operation, and the time for the patients to recover from anesthesia after the operation was recorded. Only patients with a score of 4 or more could be allowed to leave the operating room or recovery room. Patients with intraoperative hypothermia could cause prolonged recovery time of anesthesia, and the recovery time of patients after surgery was generally within 60–90 min. If the recovery time exceeded this time limit, it would be regarded as delayed recovery.

#### Incidence of postoperative wound infection and length of hospital stay

Closely observed the wound healing of patients after surgery, and recorded the number of delayed wound healing and infections, as well as the number of days the patient was hospitalized.

#### Complications

Closely observed the patient's reaction, whether there were lung infections, urinary tract infections, bedsores, deep vein thrombosis, pulmonary embolism, cerebral vascular accident, gastrointestinal and myocardial infarction, establish an adverse reaction observation table, record the adverse events and their manifestations, start time, stop time and remission reasons.

#### Satisfactions

The satisfactions of patients were surveyed by telephone or email according to a Likert scale.

### Statistical analysis

SPSS 22.0 statistical software (SPSS Inc., Chicago, Illinois) was used. Efficacy and safety analyses will be conducted according to the intention-to-treat principle using the “last observation carried forward” rule. A *P* value of less than 0.05 is defined as statistically significant with 2-sided 90% CIs.

## Results

### Results of PS matching

After PS matching, outcomes were compared for 80 well-balanced pairs of patients, respectively (No-MNIP group and MNIP group). The baseline demographic details, clinical characteristics, and relevant operative information of the 160 patients in the study groups were depicted. There were no baseline statistical differences between the No-MNIP group and the MNIP group in demographic characteristics (Table 1).

**Table 1:** Patient characteristics of propensity score-matched patient groups

Variables	All patients (N = 240)		*d*
	No-MNIP Group (n = 120)	MNIP Group (n = 120)	
Age	62.3 ± 8.3	63.1 ± 8.2	0.23
Gender (M)	64	62	0.14
Tumor state (primary/metastasis)	102/18	99/22	0.23
Duration of symptom (m)	3.3 ± 1.3	3.1 ± 1.2	0.54
Chemotherapy (cases)	62	60	0.21
Radiotherapy (cases)	24	20	0.19
Pre-SINS score	8.3 ± 3.1	8.6 ± 2.9	0.33
Operating time (h)	4.6 ± 2.1	4.8 ± 1.1	0.21
Blood loss (ml)	1230.5 ± 180.2	1250.2 ± 190.5	0.31

Variables	Marched patients (N = 160)		*d*
	No-MNIP Group (n = 80)	MNIP Group (n = 80)	
Age	61.0 ± 5.2	60.2 ± 4.9	
Gender (M)	39	42	
Tumor state (primary/metastasis)	70/10	68/12	0.02
Duration of symptom (m)	3.6 ± 1.1	3.2 ± 1.5	0.01
Chemotherapy (cases)	40	38	0.03
Radiotherapy(cases)	10	12	**0.16**
Pre-SINS score	8.5 ± 2.8	8.4 ± 3.1	0.01
Operating time (min)	4.5 ± 2.5	4.7 ± 2.5	0.08
Blood loss (ml)	1300.5 ± 200.2	1200.5 ± 180.2	0.03

Bold indicate when the results of the comparisons between the two groups were statistically significant; *d*, standardized difference

### Follow-up

This study included a 3-month follow-up period. The rectal temperature was recorded after induction of anesthesia (T1), 30 min (T2), 60 min (T3), 90 min (T4), 120 min (T5), 150 min (T6), and 1 day after operation (T7). Follow-up was completed in all 160 patients, and there were no missed cases.

### General results

There was no significant difference in the baseline VAS scores and Frankel grades in the two study groups. All patients achieved remarkable pain relief (postoperative VAS score range 0–2) and improvement in neurological function (postoperative Frankel grade D or E).

#### Evaluation of temperature changes during surgery

A comparison between the No-MNIP group and MNIP group concerning various temperatures during surgery was depicted in Table 2. Patients in the MNIP group had a significantly higer temperature as compared to the No-MNIP group at T4-7 point. (p < 0.05) The above results indicated that the MNIP had better insulation effect at 90 min, 120 min, 150 min after anesthesia induction and after surgery. (Table 2).

**Table 2:** Evaluation of temperature changes during surgery

Time Point	No-MNIP Group (n = 120)	MNIP Group (n = 120)	*P*
T1	36.89 ± 0.34	36.90 ± 0.30	0.632
T2	36.62 ± 0.45	36.91 ± 0.34	0.313
T3	36.23 ± 0.36	36.67 ± 0.36	0.329
T4	35.88 ± 0.27	36.50 ± 0.42	0.012*
T5	35.72 ± 0.28	36.44 ± 0.47	0.024*
T6	35.61 ± 0.34	36.44 ± 0.47	< 0.001*
T7	35.24 ± 0.89	36.44 ± 0.66	< 0.001*

The bold values indicated when the results of the comparisons between the two groups were statistically significant

*Indicate when the results of the comparisons between the two groups were statistically significant

#### Evaluation of anesthesia recovery effect

The results showed that there was significant difference between the two groups in the recovery time of postoperative anesthesia recovery, with statistical significance (P < 0.05). The scores of anesthesia recovery ability in the MNIP group were significantly improved, indicating that the cardiopulmonary function and circulation metabolism of patients were improved after the multi-mode insulation intervention, and the anesthesia time was significantly shortened (Table 3).

**Table 3:** Evaluation of anesthesia recovery effect

Variables	No-MNIP Group (n = 120)	MNIP Group (n = 120)	*P*
Steward score	2.75 ± 2.11	3.81 ± 1.54	0.023*
Recovery time (h)	1.12 ± 0.55	0.89 ± 0.37	0.013*
Recovery delay rate (%)	15.65%	6.76%	< 0.001*

The bold values indicated when the results of the comparisons between the two groups were statistically significant

*Indicate when the results of the comparisons between the two groups were statistically significant

#### Evaluation of incidence of postoperative wound infection and length of hospital stay

It was observed that only 5 cases of postoperative wound infection/ delayed wound healing occurred in the MNIP group, which was lower than 12 cases in the No-MNIP group. In addition, the wound infection/ delayed wound healing rate of patients in the MNIP group group was also shorter (P < 0.05), thus the result indicated that MNIP reduced the length of postoperative hospital stay. (Table 4).

**Table 4:** Evaluation of incidence of postoperative wound infection and length of hospital stay

Variables	No-MNIP Group (n = 120)	MNIP Group (n = 120)	*P*
Incidence of delayed wound healing and infections (n, %)	12, 10%	5, 4.2%	0.031*
Length of hospital stay (d)	5.67 ± 4.15	4.35 ± 3.67	0.013*

The bold values indicated when the results of the comparisons between the two groups were statistically significant

*Indicate when the results of the comparisons between the two groups were statistically significant

#### Complications survey

The summary of postoperative complications in 30 days was depicted in Table 5. There were 16 cases of complications in the No-MNIP group and 5 cases in the MNIP group postoperative, which have significant statistical difference. 5 cases and 2 cases occurred deep venous thrombosis in two groups, respectively. 6 cases and 2 cases occurred urinary tract infection in two groups, respectively. 3 cases and 1 cases occurred respiratory tract infection in two groups, respectively. 1 case occurred gastrointestinal in the No-MNIP group. 1 case occurred myocardial infarction in the No-MNIP group. No deaths, no readmission, and no reoperation occurred in either group. (Table 5).

**Table 5:** Comparison of complications between the two groups

Variables	No-MNIP Group (n = 120)	MNIP Group (n = 120)
Infection	0	0
Deep venous thrombosis	5	2
Urinary tract infection	6	2
Respiratory tract infection	3	1
Pulmonary embolism	0	0
Cerebral vascular accident	0	0
Gastrointestinal	1	0
Myocardial infarction	1	0
30-Day readmission	0	0
30- to 90-Day readmission	0	0
Reoperation	0	0

#### Satisfaction survey

Summary of the satisfaction survey was depicted in Table 5. There was no significant difference in “Overall surgical result”, “Current health status”, “Quality of cares”, “Satisfaction about LOS”, “I would redo it” and “I would advise it to a relative” scores in the two study groups, respectively. (p > 0.05) (Table 6).

**Table 6:** Comparison of satisfaction between the two groups

Variables	No-MNIP Group (n = 120)	MNIP Group (n = 120)
Overall surgical result (satisfied or very satisfied), (%)	58	64
Current health status (satisfied or very satisfied), (%)	59	62
Quality of cares (satisfied or very satisfied), (%)	60	63
Satisfaction about LOS (agree or strongly agree), (%)	50	51
I would redo it (agree or strongly agree), (%)	50	50
I would advise it to a relative (agree or strongly agree), (%)	52	50

## Discussion

There was no single intervention to prevent hypothermia that may occur during surgery. During the operation, it was often necessary to adopt a multi-mode comprehensive thermal insulation scheme, which was actively adjusted according to the dynamic changes of the patient's body temperature during the operation, so as to effectively reduce the incidence of postoperative complications and ensure the perioperative safety of patients. In order to exclude the influence of ambient temperature on the change of body temperature of patients, the operating room temperature in the control group and the comprehensive insulation group was set at 22–24 °C in this study. Intravenous heating liquid could make the patient's body temperature rise, and the effect would be better combined with other insulation measures. Heating devices for active insulation included infrared lamps, electric blankets, blankets or warm water circulating beds, hot air blowers or convective air heating systems. Air heating was the most studied intervention measure at present. It could increase the central temperature by 0.5–1 °C, which had good heating effect. It could also reduce heart complications, reduce the probability of infection and wound healing, and reduce the incidence of postoperative shivering. In this study, the incidence of hypothermia in the MNIP group was significantly lower than that in the conventional insulation group, suggesting that the insulation measures adopted in this study had good effects and could reduce the incidence of hypothermia in patients [14, 20–23].

Perioperative hypothermia could lead to increase intraoperative blood loss, prolong postoperative anesthesia recovery time, increased heart rate, increased oxygen consumption, impaired immune function, and increas postoperative complications (such as coagulation dysfunction, arrhythmia, wound infection). During general anesthesia, the threshold of hypothalamic thermoregulatory center response to hypothermia was lowered by general anesthesia. In addition, anesthetic drugs had an inhibitory effect on the central self-regulation system that maintained normal body temperature. Muscle relaxants woud block thermogenesis and hypothermia, lead to thermoregulation of vasoconstriction, reduce subcutaneous oxygen tension and cause tissue hypoxia, and indirectly inhibit the function of neutrophils, thus inhibiting the immune function of the body. Hypothermia aggravates the consumption of postoperative protein and affects wound healing, decreased platelet function, impaired coagulation function, increased blood viscosity, and even DIC, caused electrolyte disruptions that can lead to serious complications such as arrhythmias. Based on some studies, the World Health Organization issued surgical safety guidelines and clearly stated that, maintaining normal intraoperative body temperature could effectively reduce the incidence of surgical site infection (SSI) after surgery. Since then, various complications caused by intraoperative hypothermia and effective means of thermal insulation have attracted more and more attention and research in the medical field. In this study, the incidence of intraoperative hypothermia was effectively reduced by adopting the multi-mode insulation scheme, thus reducing the incidence of incision infection and shortening the length of hospital stay of patients [24–28].

At present, there were many methods of intraoperative insulation, but many effective insulation measures had not been effectively promoted due to the high price of the instrument and cumbersome operation. Natural insulation was still the main prevention measure of hypothermia in the majority of primary hospitals, and the effect of such methods was limited. In addition, with the rapid development of medical technology, more and more complex operations have been carried out in clinical practice, and the factors causing intraoperative hypothermia have become more and more complicated. How to take more effective preventive measures to reduce the incidence of intraoperative hypothermia needed further study and discussion. Taking this as the starting point, this study developed and optimized the all-round multi-mode operating room thermal insulation plan, and created a multi-mode thermal insulation plan from various processes such as environment, personnel, facilities and equipment, and operating norms, so as to provide the optimal thermal insulation for patients during operation.

## Conclusion

In this study, the incidence of intraoperative hypothermia was effectively reduced by adopting the multi-mode insulation scheme, thus reducing the incidence of incision infection and shortening the length of hospital stay of patients.

## Data Availability

The datasets used and/or analyzed during the current study are available from the corresponding author on reasonable request. Ethics approval and consent to participate. The study design, procedures, and informed consent procedure were approved by the Changzheng Hospital. Consent to participate will be obtained from the participants.

## Abbreviations

ERAS: Enhanced recovery after surgery
MNIP: Multi-mode nursing insulation program
PS analysis: Propensity score-matched analysis
